# The Challenge of Quantification: An Interdisciplinary Reading

**DOI:** 10.1007/s11024-022-09481-w

**Published:** 2022-12-21

**Authors:** Monica Di Fiore, Marta Kuc-Czarnecka, Samuele Lo Piano, Arnald Puy, Andrea Saltelli

**Affiliations:** 1 Institute for Cognitive Sciences and Technologies, Consiglio Nazionale delle Ricerche, Via San Martino della Battaglia, 44, Rome, 00185 Italy; 2 Faculty of Management and Economics, Gdansk University of Technology, Traugutta 79, 80-233 Gdańsk, Poland; 3 School of the Built Environment, University of Reading, JJ Thompson Building, Whiteknights Campus, RG6 6AF Reading, UK; 4 School of Geography, Earth and Environmental Sciences, University of Birmingham, Birmingham, B15 2TT UK; 5 UPF Barcelona School of Management, Carrer de Balmes, 132, 08008 Barcelona, Spain; 6 Centre for the Study of the Sciences and the Humanities (SVT) - University of Bergen (UIB), Parkveien 9, PB, 7805, 5020 Bergen, Norway

**Keywords:** Sociology of quantification, Metrics, Rating, Mathematical modelling, Algorithms, Ethics of quantification

## Abstract

The present work looks at what we call “the multiverse of quantification”, where visible and invisible numbers permeate all aspects and venues of life. We review the contributions of different authors who focus on the roles of quantification in society, with the aim of capturing different and sometimes separate voices. Several scholars, including economists, jurists, philosophers, sociologists, communication and data scientists, express concerns or identify critical areas of our relationship with new technologies of ‘numericization’. While mindful of the important specificities of the different families of quantification, we use our broad and holistic canvas to explore possible spaces for a more systematic investigation of incumbent and novel quantifications, as to increase communication among disciplinary communities, and among these and society, in the pursuit a democratic agency and self-defence.

## Introduction

“*Count what is countable, measure what is measurable, and what is not measurable, make measurable.*” So taught Galileo, circa 1610, and his lesson was well taken in the four intervening centuries. According to historian Alfred W. Crosby (1996), new technologies of quantification and visualization of space and time gave rise in the XIV century to a true revolution in mathematics, music, painting, accounting, astronomy, cartography and other domains. For Crosby, these changes ensured the epochal success of the West and its eventual domination over the rest of the world achieved in the space of the subsequent XV and XVI centuries^1^. At present, it appears as if this process of ‘numericization of the real^2^, ‘avalanche of numbers’ (Hacking 1990) or ‘data colonialism’^3^ (Couldry and Mejias 2019)—far from losing its momentum, is set on an accelerating pace, as shown by flourishing works and movements around the sociology of numbers (Mennicken and Espeland 2019; Mennicken and Salais 2022a; Popp Berman and Hirschman 2018), to mention just the most recent works. According to scholars, numbers are seductive and performative (Engle Merry 2016); they confer epistemic power and legitimacy (Porter 1995); they increasingly pervade (Bruno, Didier, and Vitale 2014a, b; Espeland and Stevens 2008; Saltelli 2020; Saltelli and Di Fiore 2020) and colonize (Couldry and Mejias 2019) different aspects of life. Quantification promises agency over the future and the taming of uncertainty (Scoones and Stirling 2020). It brings a class of quantifiable issues to the fore, while simultaneously leaving issues less amenable to quantification in a background. This phenomenon has become more visible in relation to what numbers should be considered to deal with the COVID-19 pandemic (Didier 2020; Miller 2022; Saltelli et al. 2020a).

For some (O’Neil 2016; Popp Berman and Hirschman 2018), the present is confronted with a blurring of the distinction between visible numbers, such as those produced by models, metrics and statistical inference, and invisible ones, such as those processed into proprietary software in artificial intelligence, big data in algorithms, hidden to users and the general public^4^. Forms of quantification as different as influencing of voters or consumers, cyberwarfare, ranking of higher education, policing, administering justice and so on, in a growing list (Zuboff 2019), have increased in scope and reach. Today, dystopian futures are being imagined in relation to old and new technologies of quantification (Muller 2018; O’Neil 2016; Supiot 2007), in their present expanded capacity made possible by more data, faster processing and a new media landscape (Saltelli and Boulanger 2019), in what has been variously defined as platform capitalism (Lanier 2006; McAfee and Brynjolfsson 2017) or surveillance capitalism (Zuboff 2019). Numbers play a key role in our sociotechnical imaginary, understood as how visions of scientific and technological progress carry with them implicit ideas about public purposes, collective futures, and the common good (Jasanoff and Kim 2015).

The voices heard includes those of sociologists, philosophers, economists, media experts, data scientists and jurists, all variously concerned with the transformative role of numbers at the social, economic and political dimensions, and with their capacity to transmit values and determine what is normal (Amoore 2020).

In the present work (Section 1) we review recent contributions on quantification and complement them with what, in our view, constitutes relevant lines of analysis. Beside the already mentioned work of Crosby (1996), surprisingly ignored by reviews of sociology of quantification, we also include in our broad canvas the debate among statisticians – veritable statistical wars on the use and abuses of inferential statistics (Mayo 2018; Stark and Saltelli 2018), the debate on the mathematization of economics (Drechsler 2000; Mirowski 2013a), and finally the discussion (or lack thereof) within the family of mathematical modelling (Saltelli et al. 2020a). We call this canvas the “multiverse of quantification”, to highlights that many of the authors and actors reviewed inhabit different worlds.

While Mennicken and Espeland (2019) caution against unifying accounts of quantification, and highlight the importance of tracking quantification across different sites, we explore (Section 2) strategies and spaces for democratic agency and collective self-defence that might be seen as common to the various regions of the multiverse.

## Section 1. Voices from Different Disciplines

We give in this section the voices from different disciplines. Occasionally we flag where these strands don’t talk to each other, or areas of high societal relevance that are less investigated by academic scholarship. If the section appears cacophonic, rather than polyphonic, this is because of the nature of the landscape.

To be noted, the analysis of the excesses in quantification offered in the present section is by no means a refutation of quantification. The critique of the economic assessment and of the occasionally implausible cost-benefit analysis found here does not imply that economic analyses are systematically biased or wrong^5^. On the same ground, the critique of disciplines such as evolutionary or cognitive psychology does not imply that all contributions from these disciplines are damaging^6^. The critique of the present section targets excess not application, abuse and not use, the generation of knowledge asymmetries not the process of social discovery.

### ***Cartesian Anxiety: Numbers, Uncertainty, Ignorance***

Some scholars (Reinert et al. 2021) propose a way to understand the penetration of numbers in all venues of life, and the faith in their logical assumptions, going back to the *Cartesian Dream* of certainty, neutrality, and control of man over nature made possible by natural philosophy (Davies and Hersh 1986; Pereira and Funtowicz 2015; Toulmin 1992). This dream starts with Francis Bacon and William Petty in the XVII century, to continue with Condorcet’s Mathématique sociale (Feldman 2005), continues through ages e.g. via Bentham’s utilitarianism in the XIX century, and the post-war decisionism inspired by the successes of operational research during World War II (Majone 1989), and reaches a high point with the prevalence of New Public Management theories in both private and public sectors started in the eighties (Mennicken and Salais 2022a).

‘Decisionism’, the idea that decisions can always be systematically arrived at via computation, has powered a ‘procedural utopia’, assuming the existence of machinery to take the right decision based on a set of logical rules and methods (Millgram 2015). Andrew Stirling (2019), commenting on the use of terms such as ‘expected utility’, ‘decision theory’, ‘life cycle assessment’, ‘ecosystem services’ ‘sound scientific decisions’ and ‘evidence-based policy’ notes that “Each technique routinely delivers its answers with formidable levels of precision. Yet the resulting impression of accuracy is deeply misplaced^7^”. In a subsequent multi-authors volume, Scoones and Stirling (2020) offer many relevant examples of compression of uncertainty in policy evaluations via technologies of quantification. The modern apparatus of computation does away with uncertainty and ambiguity, aiming to reduce them to (Knightian) risk, and, as in the case of financial mathematics, colonizes the future, transforming it into an occasion for profit in the present (Walter and Wansleben 2020).

### ***Numbers and Quantification: Different Takes***

In his classic work, Porter (1995) analysed the appealing aspects provided by numbers, such as trust, authority and legitimacy in a decision-making process. Numbers seem fair and impartial, and their “objectivity lends authority to officials who have very little of their own”. The purported objectivity of numbers (Daston 1992) can be used to deflect contestation. This may enhance knowledge and power asymmetries, as “Fighting a number with a number”, as per the motto of statactivists (Bruno, Didier, and Prévieux 2014; Bruno, Didier, and Vitale 2014a, b), requires resources and infrastructures not available to the lay citizen (Mennicken and Espeland 2019; Salais 2022; Samuel 2022).

Numbers affect other numbers, they generate lock-in and path dependencies, which are resilient to change, due to what sociologist Sally Engle Merry (Engle Merry 2016) defined as “expertise inertia” and “data inertia”. Incumbent numbers affect whom, what and how society will count and will measure in the future. Moreover numbers generate “reactivity” (Espeland and Sauder 2016) among those who are subject to them, possibly generating “pernicious feedback loop[s]” that “create the environment that justifies their assumptions” (O’Neil 2016).

Mennicken and Salais (2022a) in their introduction to the volume ‘The New Politics of Numbers’ offer an agile introduction to the Foucauldian studies of governmentality - which first flourished in the English-speaking world, and to the studies of state statistics known as ‘Economics of Convention’, developed in France, mostly at INSEE - Institut National de la Statistique et des Etudes Economiques (the French Statistical office), thanks to Alain Desrosières, Laurent Thévenot and the same Salais. The title of the volume of Mennicken and Salais points to Desrosières' (1998) own work ‘The Politics of Large Numbers’, an important text on the genesis of probability and statistics^8^. Also, to be mentioned in the French school is sociologist Luc Boltanski, collaborating with Thévenot on grammars of “Orders of worth” that are needed to make sense of how justice is itself justified by different actors under different normative frames. So market-worth is distinguished from civic-worth, industrial-worth, and others (Boltanski and Thévenot 2006; Thévenot 2022)^9^.

In an early (2008) review of sociology of quantification, Espeland and Stevens observed the increasing political demand for *social numbers* and the emergence of what they call new regimes of measurement. Already ten years after, Popp Berman and Hirschman (Popp Berman and Hirschman 2018), portrayed a changing landscape, where “a proliferation of scholarship on numbers goes hand in hand with a proliferation of numbers itself”. Here, the quantified-self and big data are the incumbent challenges due to the new technologies and the explosion of internet services. Berman and Hirschman observe that the spread of numbers has reinforced the “regimes of measurement” and has empowered different actors who co-shape the production and governance of numbers. A recent review of the field over the four domains administration, democratic rule, economics, and personal life are concerned (Mennicken and Espeland 2019) confirms these trends. We do not have the ambition to reproduce here Mennicken and Espeland’s thorough review, inclusive of a history of numbers in the four domains. We focus on some elements we need in our analysis and add others from our own readings, especially in the dimension of statistical and mathematical modelling not covered by these authors^10^.

For Shoshana Zuboff (Zuboff 2019), the extraordinary development of algorithms and big data has permitted to the owner of major platforms to conduct real time experiments of cognitive psychology at an unprecedented scale, endowing these actors with a new “instrumentarian power” of knowing and orienting the behaviour of consumers and voters in a way which challenges the prospects of democracy.

The critique of the jurist Alain Supiot (2007) focuses on the uses of economic and social quantifications in the pursuit of ideas of efficiency and competitiveness in a global market. In his ‘Governance by Numbers’, Supiot contrasts a society governed by just laws with one governed by numbers. For this author, we have entered the era of the cybernetic imaginary, which revives the West's age-old dream of grounding social harmony in calculations – the ‘Cartesian Dream’ just mentioned. According to Supiot, the term cybernetics refers to the fact that, following the theories of New Public Management, workers act to achieve predefined goals; in this post-Taylorian arrangement, salaried people no longer sell their hours of working time but subscribe to a regime of permanent mobilization in the pursuit of assigned objectives.

This new regime leaves no option for populations or countries than to ride roughshod over social legislation, and pledge allegiance to those stronger than they are. In the pessimistic vision of Supiot, this amounts to a technology-driven (re)feudalization of society, as protection can only be obtained by a most powerful agent.

The issues of democracy in decision-making processes led by numbers (Supiot 2007; Zuboff 2019) have led to a new field of investigation: algorithmic governmentality, extending both quantitatively and qualitatively the scope of the classic use of statistics in the administration of a state (Rouvroy and Berns 2013). As noted by several scholars, a decision achieved by an algorithm evades a process of negotiation and deliberation, eliminating even the existence of an appealable trace (Brauneis and Goodman 2018).

The clearest illustration of the complex relationship between measures and policies is offered by Robert Salais (2022) in his “La Donnée n’est Pas Un Donné’: Statistics, Quantification and Democratic Choice”. Here Salais illustrates the progressive ‘unmaking’ of statistics over the past four decades to achieve what he calls “governance driven quantification”. Following Desrosières (1998), Salais presents ‘statistics’ as the activity of creating through the categorization and attribution of statistical objects that work in the definition and analysis of social problems, what we could call the basis for evidence-based policy, (see Table 1).

**Table 1:** The changing relationship between measures and policies

**Evidence-based policy**	Statistics: creating statistical objects that hold together for the solution of practical problems (Desrosières, 1998).
**Policy-based evidence**	Governance-driven quantification: a reversal of the statistical pyramid (Salais, 2022), where the measure is selected as to confirm the effectiveness of policy.

Conversely for Salais “Governance-driven quantification” represents a “reversal of the statistical pyramid”, where the design of the measures follows the logic of proving that an objective is both desirable (justificationism) and achieved (policy-based evidence). The example offered by Salais concerns employment policy, where the achievement of an objective, the maximization of employment rate, is pursued while emptying the meaning of employment, and hiding precariousness and insecurity. In the same volume (Mennicken and Salais 2022a), Ota De Leonardis (2022) develops a similar analysis for the case of inequality. Here De Leonardis shows how quantification of poverty and inequality were accompanied by a semantic shift obscuring the bonds of domination linking the subjects, and where inequality becomes “a distributive difference, a gap, a disparity: a distance, and no longer a tie”, realizing what she calls the dreams of “indifferent power”, shifting out of focus important dimensions of power, politics, and institutions. In the same volume, (Thévenot 2022) offers an illuminating analysis of how quantification, as deployed in the setting of standards, helps to transform common goods – such as collective solidarity, environmental concerns and the role of traditions into commercial labels. In this way, civic worth, green worth and domestic worth are subsumed under the category of market worths.

For data scientist Cathy O’Neil, models, indicators or algorithms used in decision-making have three attributes which can make them dangerous as “Weapons of Math Destruction”: they are opaque, damaging, and scalable (O’Neil 2016). Scalability here refers to the ease with which different form of quantification via visible (ranking) or invisible (algorithms) numbers can be scaled up both in geographically, from the local to the global, and across different sectors. As discussed in the conclusions, O’Neil’s work played an important hinge work between academia, society, and media.

### ***Finance and Economics***

Finance and economics (Drechsler 2000; Reinert 2000; Romer 2015) are perhaps the fields where the dangers of mathematization have been more visibly discussed, also in relation to the onset of the last recession (Porter 2012; Ravetz 2008; Wilmott and Orrell 2017). Mirowski (2013b) criticizes the normative propensity of mathematical models in macroeconomics. Romer, the former chief economist of the World Bank, argues that economic models are tools for regulatory choices, values and interests, hidden under a veil of mathematics (Romer 2015). For (van Zwanenberg 2020), economic quantification is linked to a form of technocratic orthodoxy, and to reductionism in the framing of human and social affairs. When the scientific community joined the campaign “Doing Rights Not Rankings”, A relevant form of *statactivism* (though not under this name) emerged. With the support of over 360 signatories from 80 countries, activists called the World Bank and its shareholders to end the Ease of Doing Business rankings (Ghosh 2020) for its perverse effect on social policies in developing and developed countries.

### ***Opening the Disciplinary Boxes***

The existing literature still lacks ‘general claims or a common theoretical language’, ‘a well-defined object of study’ and a connection among studies on quantification across different disciplines (Popp Berman and Hirschman 2018). In fact, sociology of quantification still appears as “a genre, not a subfield” (*ibidem*), although Mennicken and Espeland (2019) point out that this lack of connection is not necessarily a “bad thing”, given the specificities of different practices.

If one accepts that existing sociological studies of quantification, while precious in their own way, are nevertheless fragmented^11^, then strengthening the connection among studies on quantification across different disciplines and subfields become both a necessity and an opportunity for multidisciplinary scholarship. The opening of the disciplinary boxes would allow escaping both “data inertia” and “expertise inertia” mentioned by (Engle Merry 2016).

A worthy topic would be the quest for generalized quality assurance rules and the opening up of the entire process of quantification, including the underlying framings, assumptions, data, narratives, interests and stakes. This evidently calls for better dialogue between that two great science families^12^ – creating an agora where the mathematical and the ethical could be tackled side by side. The idea of an ethics of quantification, introduced by Espeland and Stevens (Espeland and Stevens 2008), appears promising, though not of easy application. Take for example algorithms: a common refrain in the present discourse of ethics of algorithms is that these should be corrected to become ‘good’ or ‘fair’ or at least ‘transparent’. A main difficulty with this approach is that algorithms are not developed for this purpose. No bank would adopt a profiling software that would give money to customers with no money. Moreover, as Amoore (2020) discussed, an algorithm is an “ethicopolitical arrangement of values, assumptions, and propositions about the world”. Once put in operation, the algorithm creates a new reality, with new practices, new norms of good or bad. As a clue of the ethical authority invested in algorithms, AI experts are actively investigating ethical machines, called artificial autonomous moral agents (AAMAs), to function as a moral prompter (Lo Piano 2020), e.g. in the taking of medical decisions (Anderson and Leigh Anderson 2007). Otherwise said, technologists’ dreams to ‘moralize’ algorithms contrasts with the fact that what is moral is increasingly shaped by algorithms (Amoore 2020).

Going back to a possible ethics of quantification as a vehicle for shared criteria of quality, Amartya Sen’s Informational Basis for Judgment of Justice (IBJJ) (Sen 1990) suggest looking at quality along two axes: that of technical adequacy – for which presumably each family of quantification disposes of its own criteria, and that of fairness. Sen’s framework, also recommended by (Salais 2022), suggests to explore whether a given measure permits a fair, informed judgement of an issue. Fairness implies that a measure should weight the chances of individual to achieve their goal, in a way that is mindful of the individual condition (capability approach). Therefore, fairness does not correspond to equal material means for all, but to equal opportunities for all to fulfil one’s aspirations.

### ***Mathematical Models and Statistical Controversies***

There are two aspects why mathematical modelling, intended *sensu lato* as to cover different kinds of analytic constructs, could be central to an ethics of quantification. The first is that mathematical modelling is pervasive, part and parcel of practically all quantification activities – from algorithms to ratings to the making of aggregate measures such as the now extremely popular family of composite indicators (Kuc-Czarnecka, Lo Piano, and Saltelli 2020). Modelling is also central to the use of statistics. Yet, in the field of statistics momentous changes have taken place. After years of soul-searching due to the poor use of statistical inference, the so called-reproducibility crisis in science (Saltelli and Funtowicz 2017) has pushed the discipline of statistics at the forefront of the controversy (Stark and Saltelli 2018). Statistical institutes urgently issue guidelines (Wasserstein and Lazar 2016), while a significant number of statistical professionals launch a petition to abolish the concept of significance altogether (Amrhein, Greenland, and McShane 2019; Gelman 2019). Statistical wars are now part of the new normal (Mayo 2018).

While statistics sorts in the daylight what might constitute a set of norms for responsible inference, this situation is rather more obscure in the field of mathematical modelling, which is not a discipline by itself. Thus different modelling communities are ‘united’ by a lack of standardized quality control procedures (Padilla et al. 2018). The extreme dependency of the inference by apparently inconsequential modelling choices never cease to surprise the same experts (Breznau et al. 2021; Frigg et al. 2014).

A recent manifesto on mathematical modelling highlighted the problems in relation to COVID-19 and the potentially dangerous role of model-generated numbers in the pandemic (Saltelli et al. 2020a). The manifesto stressed that modelling is a social activity–but the same can be said of any form of quantification, whose proper use demands that producers and users of numbers come to domesticate one another to the effect that numbers help rather than harm.

The five principles proposed in Saltelli et al. (2020a) cover the transparency of assumptions, the proportionate use of modelling, the attention to the context of use, and the consequences of a quantification, and finally a Socratic respect for ignorance.

The literature has discussed the opportunism of the so-called “chameleon models” (Pfleiderer 2020), alternatively presenting themselves as tools for political prediction or as theoretical analysis according to the opportunities, taking refuge in the second role as they are caught out in the unwarranted exercise of the first, e.g. when an undocumented research code is used as a policy tool.

A present discussion is what part of models used in determining COVID-19 policies belong to this class (Saltelli, Bammer, et al. 2020b). The same source (Saltelli, Bammer, et al. 2020b) also wonders how concepts such as the value of a statistical life might have a place in choosing the best public-health policy. Since this metric can produce an appearance of rigour, it also runs the risk to disguise political decisions as technical ones, as shown by a recent debate about the depoliticizing tendencies of quantification (Mennicken and Espeland 2019). That numbers may have a de-politicizing tendency - used instrumentally by various actors, is indeed not new, and has been a recurring theme flagged by ecologists, e.g. in relation to risk or cost benefit analyses, see chapter 8 in (Winner 1989) and by scholars of science and technology studies from Daston (1992) to Jasanoff (2003).

The government by numbers has also created a system of *networking of numbers*, reinforcing the concept of *inertia* discussed by (Engle Merry 2016), and creating a meta- or second-order measurement that enables new forms of comparison and knowledge creation (Power 2004). Then, these numbers ‘take on a life of their own and are circulated and removed from their origins of production’ (Mennicken and Espeland 2019). Ranking of higher education might be seen as an example of this tendency, whereby a tool developed for local needs becomes global and every dean of the worldwide education system has to follow its indications (Éloire 2010).

## Section 2. What Spaces for Democratic Agency?

Some authors have recently suggested the possibility of an observatory for ethics of quantification (Saltelli et al. 2021).

A few examples of ‘missions’ that could be entrusted to an observatory for ethics of quantification are given below.

- Mathematical models can be subject to a regime of investigation deploying tools such as sensitivity auditing (Saltelli et al. 2013), an extension of sensitivity analysis recommended for models to be used for policy (Science Advice for Policy by European Academies 2019). Similar concerns inspire the use of pedigrees in quantitative information, such as NUSAP (Funtowicz and Ravetz 1990; van der Sluijs et al. 2005), or the appeal of Sheila Jasanoff for technologies of humility against technologies of hubris (Jasanoff 2003).
- “Algorithms of public relevance” (Gillespie 2014) are those with socio-political impacts. How to identify them? What to do once they are identified? A relevant example is offered by O’Neil of ‘hackathons’ to reverse-engineering proprietary software and to identify normative or political bias (O’Neil 2016).
- Given the prevailing narrative whereby numbers play as an assurance of neutrality prediction and control, a resistance should be based on unveiling the non-neutrality of numbers and methodologies (Saltelli et al. 2020a, b, c) and their underlying, spoken and unspoken, frames and assumptions (Saltelli et al. 2013). This line of activity would go in the direction of contrasting numerical hubris with a ‘culture of humility’ (Jasanoff 2003).
- The neglect of ambiguity (Gupta 2001) and of ‘not-knowing’ limits the space of the possible policy solutions. As noted for the case of COVID-19, science-based, number-based policies may offer politicians the opportunity to abdicate decisions by transforming a political decision into a technical one.
  *Modellers must not be permitted to project more certainty than their models deserve; and politicians must not be allowed to offload accountability to models of their choosing* (Saltelli et al. 2020a).
- As mentioned, several communities have attempted to reform the production of numbers (Algorithmic Justice League 2020; Bruno, Didier, and Prévieux 2014; Cardiff University 2020; French National Research Institute for Sustainable Development 2020). These communities may benefit from further spaces, programs and synergies to achieve major impact.
- Since those with the deepest pockets can purchase the most evidence and disseminate it more efficiently (Drutman 2015; Laurens 2017; Saltelli 2018; Saltelli et al. 2020a, b, c), policing in which numbers populate the public arena is also a way to be active in these power games. As argued in (Foucart, Horel, and Laurens 2020; Saltelli et al. 2020a, b, c) the sophistication of the strategy played by private interests are ever increasing, including occupying spaces created by well-meaning participatory strategies (Mirowski 2020).
- Alternative measures to quantitative metrics to break the exclusivity of metric regimes.

## Conclusion

The present work and the literature it interrogates has examined the critical aspects of quantification. Yet these voices most likely constitute the view of a minority. In the present prevailing imaginary, the methodologies and technologies of datafication provide humanity with unprecedented means to tame uncertainty and rule human affairs (Pinker 2018; Sunstein 2020)^13^. Coupled with nanotechnology, quantification with artificial intelligence will allow a new “Fully Automated Luxury Capitalism” according to Bastani (2019). Though a ‘tongue in cheek’ use of the terms can be suspected here, the book reads as a serious run through technologists’ aspirations, e.g. to mine asteroids for precious minerals. Some intellectuals appear to consider the risk from datafication remote, and at most in the risk of a future ‘digital dictatorship’ or that of ‘hackable humans’, this latter posed by convergent AI and nano technologies (Harari 2018)^14^, against those who see such a dictature already in progress (Salais 2022; Supiot 2007) and the humans already hacked (The Social Dilemma 2020; Zuboff 2019).

For technologists McAfee and Brynjolfsson, our digital future needs to be harnessed (McAfee and Brynjolfsson 2017). In Europe, a digitalization agenda pervades European research, e.g. in the European Union Horizon Europe programme, to address environmental as well as social and health problems – see the concepts of ‘green and digital transitions’ and ‘digital twins’ (European Commission 2020). It appears that–as with many contemporary issues, such as frontier technologies, e.g. transhumanism^15^, one side is mesmerized by the potentialities of what another side considers an impending dystopia.

In our analysis, we have focused on both visible and invisible numbers, what we call “the multiverse of quantification”. Visible numbers densely populate the science policy interface, whereby no branch of government nor strand of public life escapes the use of numbers to adjudicate priorities. Distortion and abuses of quantification are well documented in the literature reviewed in the first section of the present work.

As per the invisible numbers, those of the algorithms, that are either produced by the users themselves or collected by platforms, institutions and other societal actors are coerced into submission by the existing knowledge asymmetry caused by the opacity of the algorithms, and by the speed of transformation whereby a new normal established itself (The Social Dilemma 2020).

The existing *regimes of measurement* are the target of the activities of the proposed observatory for the ethics of quantification. The observatory can be seen as a dialectical opportunity between the depoliticizing tendencies of quantification and the need to re-politicize them as seen in old and new initiatives (Algorithmic Justice League 2020; Bruno, Didier, and Prévieux 2014; Cardiff University 2020; *Coded *Bias 2020; French National Research Institute for Sustainable Development 2020; Radical Statistics Group 2020).

Note also that if academia’s work were to be driven by an ideal societal demand, many aspects of bad pharma linked to misuse of quantification (Harris 2017; Ioannidis 2016), or the high cost of adolescents’ lives lost to suicide apparently driven by the new media (The Social Dilemma 2020; Twenge et al. 2018), should receive more attention by academic work. We make these remarks to suggest that, while generous, the new wealth of scholarship in the domain of quantification may not be as impactful as society would need it to be, to avoid what Tristan Harris, a former design ethicist at Google, calls “checkmate on humanity^16^” (The Social Dilemma 2020).

Interdisciplinary work can help to bridge scholarship with society. ‘Coded Bias’ (*Coded *Bias 2020) is a documentary on how the fight against facial recognition software gained momentum. ‘The Social Dilemma’ (2020), is also a documentary, narrated from the inside of technologists’ world, of the damage brought about on social and political life by the new media. More people have watched these productions^17^ than will ever read Desrosiéres, Zuboff or Supiot. Even ‘popular’ works such as O’Neil (2016), Muller (2018) or Lanier (2018; 2006) reach more readers than strictly academic productions, and help projecting academia outside academia–for example, the activist behind Coded Bias was inspired by O’Neil.

Our work is more about bridges across disciplines than between academia and society. Mennicken and Espeland (2019) conclude their work noting:

The challenge remains, however, for scholars of quantification to find each other, and this will always demand breadth in reading and, perhaps, articles like this.

We can borrow, perhaps, the same closing line.

## Footnotes

1 The theories explaining the success of the West are so numerous as to constitute a ‘genre’, see e.g. (Diamond 1999).

2 Crosby’s book is entitled “The Measure of Reality”. We and other authors also use expressions involving reality becoming numerical, but this does not confirm nor denies any specific realist or anti-realist perspective. One of the distinctive features of Desrosières’ (1998) account of the genesis of statistical thinking is the refusal to take side for a purely realist or purely constructivist view of quantification.

3 Couldry and Majas (2019) use here colonization not in a North-South meaning, but in that of private spaces of existence, such as leisure time, being increasingly occupied and measured by platforms.Query

4 Even in the specific field of mathematical models, one talks of ‘data-laden models, model-filtered data’, pointing to the end of the separation between the two key ingredients of quantification (Edwards 1999).

5 To make an example from today’s post-Brexit debates, while the participation in a program such as the EU’s Erasmus is not only a matter of gain or losses, the ongoing discussion in the UK of whether money was gained or lost by exiting the program plays a legitimate role in the public discourse (Raphael 2021).

6 As an example, the role of cognitive psychology in investigating the human origins of mathematics (Lakoff and Núñez 2001) is remarkable.

7 In other words, offering results with implausible number of digits (high precision) conveys in the reader an impression of corresponding accuracy (closeness to the object measured).

8 This is not the subject of the present work, but important readings on the emergence of the concept of probability are from Hacking (e.g. 1990) and Daston (e.g. 1988). A thorough review is offered in the work of Desrosières already mentioned (1998).

9 In this framework, New Public Management can be interpreted as a push for the adoption of a market work against a civic one, see later in this work.

10 An exception is the volume of (Morgan and Morrison 1999). This work highlights the extraordinary versatility of models both as instruments to do things and as representations to reveal things, to perform a vast array of tasks. In discussing “Models as mediators” these authors argue that models are partly independent from both the theory and the world they represent: “models are not situated in the middle of a hierarchical structure between theory and the world”, p. 17. In this nature of model is the reason of their being pervasive.

11 The case of the domains of statistical inference and mathematical modelling – so close and so different in their level of analysis, is particularly relevant (Saltelli 2019).

12 To make an example, the theories of sociologist Luhmann could be usefully applied to the field of indicators of development (Boulanger 2018).

13 For a critique of Pinker and Sunstein, see respectively (Gray 2018) and (Timms 2019).

14 The example of Harari suggests that a characteristic of techno-optimism is that of situating present dangers into the future.

15 Hailed by Harari in Homo Deus (Harari 2016), trans-humanism is feared by Lent and others to produce a techno split of humanity between an affluent super-technological and possibly immortal minority, versus a useless and distracted majority left glued to its mobile phones and tablets (Lent 2017; Tintino 2014).

16 The full quote is “If technology creates mass chaos, loneliness, more polarization, more election hacking, more inability to focus is the real issues, we are toast. This is checkmate on humanity” (The Social Dilemma 2020).

17 More than 0.1 billion viewers for The Social Dilemma after https://www.humanetech.com/the-social-dilemma, while Coded Bias collected several cinematographic awards, see https://www.imdb.com/title/tt11394170/awards/?ref_=tt_awd.
